# Physicians Visiting List for ’88

**Published:** 1887-10

**Authors:** 


### BIBLIOGRAPHY.

---

THE PHYSICIAN VISITING LIST FOR 1888. (Lindsay and Blakiston's.) Thirty-seventh year, 1851—1888. P. Blakiston Son & Co., 1012 Walnut street, Philadelphia, publishers.

As usual P. Blakinston Son & Co., are the first in the field. Strength, compactness, convenience, and durability are the essential qualities which a good visiting list should possess to resist the unusual hard wear it receives. These qualities are all combined in Lindsay & Blakiston's Physician's Visiting List, which has now been published for thirty-seven years. It is the most convenient for the pocket. Its contents are arranged in the most advantageous way.

